# Baseline correction of phase-contrast images in congenital cardiovascular magnetic resonance

**DOI:** 10.1186/1532-429X-12-11

**Published:** 2010-03-05

**Authors:** Brian J Holland, Beth F Printz, Wyman W Lai

**Affiliations:** 1 Morgan Stanley Children's Hospital of New York Presbyterian, Columbia University Medical Center, New York, New York, USA

## Abstract

**Background:**

One potential source of error in phase contrast (PC) congenital CMR flow measurements is caused by phase offsets due to local non-compensated eddy currents. Phantom correction of these phase offset errors has been shown to result in more accurate measurements of blood flow in adults with structurally normal hearts. We report the effect of phantom correction on PC flow measurements at a clinical congenital CMR program.

**Results:**

Flow was measured in the ascending aorta, main pulmonary artery, and right and left pulmonary arteries as clinically indicated, and additional values such as Qp/Qs were derived from these measurements. Phantom correction in our study population of 149 patients resulted in clinically significant changes in 13% to 48% of these phase-contrast measurements in patients with known or suspected heart disease. Overall, 640 measurements or calculated values were analyzed, and clinically significant changes were found in 31%. Larger vessels were associated with greater phase offset errors, with 22% of the changes in PC flow measurements attributed to the size of the vessel measured. In patients with structurally normal hearts, the pulmonary-to-systemic flow ratio after phantom correction was closer to 1.0 than before phantom correction. There was no significant difference in the effect of phantom correction for patients with tetralogy of Fallot as compared to the group as a whole.

**Conclusions:**

Phantom correction often resulted in clinically significant changes in PC blood flow measurements in patients with known or suspected congenital heart disease. In laboratories performing clinical CMR with suspected phase offset errors of significance, the routine use of phantom correction for PC flow measurements should be considered.

## Background

Phase-contrast (PC) images are used to quantify blood flow in cardiac magnetic resonance (CMR) by measuring the phase shift of moving protons [1,2]. PC flow measurements have been shown to accurately quantify blood flow in subjects with structurally normal hearts and in those with congenital heart disease (CHD) [1,3,4]. There are a number of potential sources of error in PC CMR flow measurements, including aliasing due to inappropriate VENC parameters, signal loss due to complex or turbulent flow, partial volume averaging due to limited spatial resolution, signal misregistration due to in-plane movement during signal acquisition, and phase offset errors due to local non-compensated eddy currents [2,3,5,6]. Most of these sources of potential error can be minimized by optimizing imaging parameters. Correction of phase offsets, however, requires analysis of stationary tissue to serve as a baseline reference for zero velocity [1,2,5,6].

We designed this study with the following objectives: 1) to assess the effect of phantom correction on PC flow measurements in patients referred to a busy congenital CMR program, 2) to assess the relationship between blood vessel size and the magnitude of change in flow measurement with phantom correction, and 3) to assess whether patient diagnosis impacts on the magnitude of phantom correction. We assessed the effect of background correction using prospectively defined measures of clinically significant and marked errors.

## Methods

### Patients

We conducted a retrospective review of all patients referred to the Morgan Stanley Children's Hospital of New York Presbyterian for clinical CMR from May 2008 to December 2008. CMR data from patients who had examinations performed with PC images using phantom correction were analyzed. The decision to perform PC images was based on clinical and technical considerations for each patient

To assess the effect of phantom correction on pediatric patients with structurally normal hearts, a subset of patients with no shunts and no valvular regurgitation was identified. To assess the impact of a given diagnosis on the magnitude of phantom correction, our largest single diagnostic group, TOF, was analyzed separately.

### Image Acquisition

Examinations were performed on two identical GE TwinSpeed Signa HDx 1.5T v. 14 scanners using commercially available coils (GE Healthcare, Milwaukee, Wisconsin). Correction for concomitant gradient effect, built into the image reconstruction, was used throughout. Non-breath-hold PC images were acquired perpendicular to the vessel of interest using orthogonal long-axis views of the vessel with the commercially resident FastCine PC pulse sequence. The coil and patient were positioned so that the heart was at isocenter, and the couch move to z = 0 facility was used. Vessels were imaged near isocenter. The following parameters were used: the TwinSpeed gradient system was in the high speed mode, with small fields of view (FOV) of 25-35 cm, matrix 256 × 192, time per cine frame 33 msec, slice thickness 5 mm, TR 12.2 msec, TE min full (4.5 msec), flip angle 25°, bandwidth 15.63 Hz, and velocity encoded (VENC) 200-400 cm/s, based on clinical parameters. No additional turbo factor was used. The number of views per segment varied between 2 and 8 based on heart rate. The acquisition length was usually 90-120 seconds, with NEX = 3. Retrospective ECG gating was performed with continual gradient operation. Flow compensation was used throughout. Immediately after the patient was removed from the scanner, a stationary fluid filled phantom was moved into position, and in approximately one to two minutes the images were repeated using the same clinical parameters to establish a baseline of zero velocity. The phantom data was acquired within 15 to 20 minutes of obtaining the PC flow imaging. The size of the phantom was chosen based on patient size, and a heart rate simulator was set to 60 or 120 beats per minute. As imaging was acquired using retrospective ECG gating, the background offset was likely to be stable during cine frames, and setting the HR simulator at 60 vs. 120 beats per minute was unlikely to affect the velocity offset.

**Figure 1:**
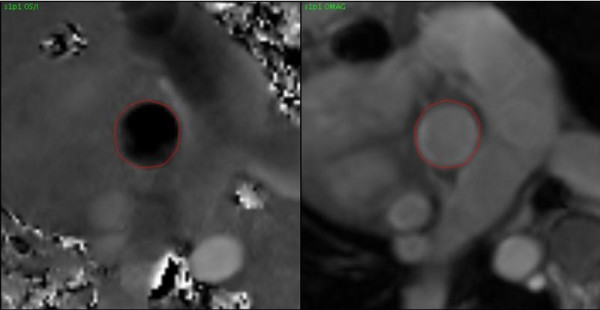
**Phase Contrast Images of the Ascending Aorta with Region of Interest**.

**Figure 2:**
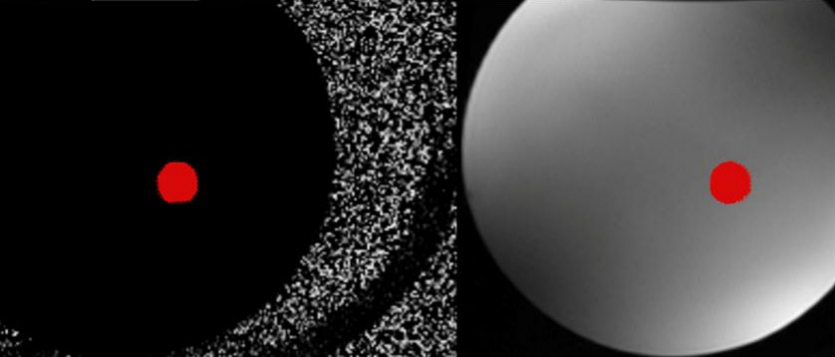
**Phase Contrast Images of the Phantom with Region of Interest from Ascending Aorta**.

**Figure 3:**
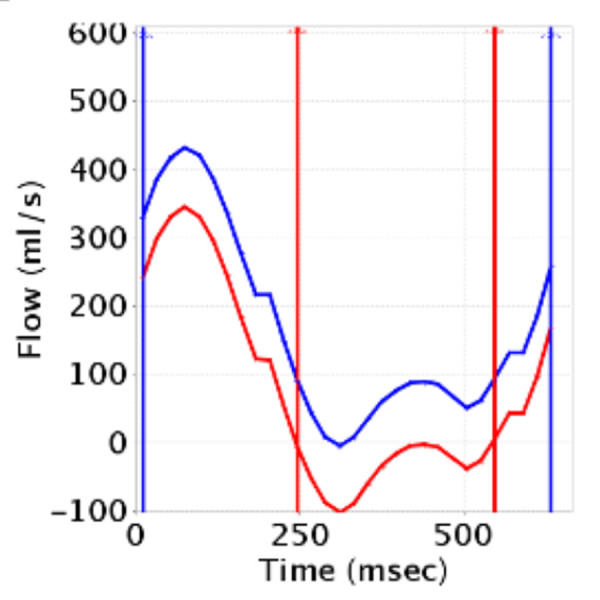
**Phase Contrast Flow vs. Time Curves**. Flow versus time curves from one cardiac cycle for the main pulmonary artery without (blue) and with (red) phantom correction. Flow volumes were calculated by integrating the area under the curves. With phantom correction, the flow in the main pulmonary artery changed from 5.1 to 2.4 L/min/m^2^, and the regurgitant fraction changed from 0% to 19%.

Based on the clinical protocol, PC images were obtained in the AAO, MPA, RPA, and/or LPA. PC images were analyzed using GE ReportCARD^© ^3.6 both with and without phantom correction (Figures 1, 2, 3). A region of interest (ROI) was drawn for each vessel, and net flow rate (ml/min) was calculated for each. Flows were corrected in the AAO, MPA, RPA, and LPA by applying the corresponding baseline shift that zeroed the flow in the phantom using the reporting software. Subtraction was done based on an average baseline rather than on an image-by-image basis, to minimize signal-to-noise ratio loss. The Q_P_/Q_S_, percent flow to the RPA (Q_P_R fraction), pulmonary regurgitation fraction (PR), and aortic regurgitation (AR) fraction were also calculated.

### Definitions

Changes in flow measurements with phantom correction were defined prior to analysis as follows, as agreed upon by consensus of two pediatric cardiologists experienced in CMR (WL, BP): (Table 1): a change in MPA or AAO flow ≥ 0.5 L/min/m^2^, change in RPA or LPA flow ≥ 0.25 L/min/m^2^, change in Q_P_/Q_S _≥ 0.4, change in Q_P_R fraction ≥ 10%, and change in PR or AR fraction ≥ 10%. These definitions were based on generally accepted normal ranges for the measured and calculated values. For example, a Q_P_/Q_S _cutoff of 0.4 was chosen as a clinically significant change because a Q_P_/Q_S _of 1.0 is generally considered to be normal while a value of 1.4 or greater is considered a potentially significant shunt. Marked changes in flow measurements for the purpose of this analysis were defined as double the amount of clinically significant change in each category--for example, a change in MPA flow ≥ 1.0 L/min/m^2 ^or PR fraction ≥ 20%.

**Table 1:** Definitions for Magnitude of Change

Variable	Clinically Significant Change	Marked Change
**MPA or AAO flow**	≥ 0.5 L/min/m2	≥ 1.0 L/min/m2

**RPA or LPA flow**	≥ 0.25 L/min/m2	≥ 0.5 L/min/m2

**Q_P_/Q_S_**	≥ 0.4	≥ 0.8

**Q**_P_**R fraction**	=≥10%	≥ 20%

**PR or AR fraction**	≥ 10%	≥ 20%

AAO-Ascending Aorta, AR-Aortic Regurgitation, LPA-Left Pulmonary Artery, MPA-Main Pulmonary Artery, PR-Pulmonary Regurgitation, Q_P_-Pulmonary blood flow, Q_S_-Systemic blood flow, Q_P_R-Right pulmonary blood flow as a percent of total pulmonary blood flow, RPA-Right Pulmonary Artery

### Statistical Analysis

Statistical analysis was performed using SPSS 16.0 (SPSS Inc., Chicago, Illinois) and Microsoft Excel 2007 (Microsoft Corporation, Redmond, Washington). The relationship between blood vessel cross sectional area and change in PC flow measurement, and the relationship between heart rate and change in PC flow measurement, were both analyzed using linear regression. For this group, the absolute difference between the measured Q_P_/Q_S _and the assumed true Q_P_/Q_S _of 1.0 were compared with and without phantom correction using paired t-tests. To assess the impact of a given diagnosis on the magnitude of phantom correction, our largest single diagnostic group, tetralogy of Fallot, was analyzed separately. A chi-squared test was used to compare the subgroup with tetralogy of Fallot with the entire group of analyzed patients, and to compare the measurements that were increased or decreased following phantom correction. A p value of < 0.05 was considered statistically significant.

## Results

### Patient Characteristics

There were 149 patients identified who had clinical CMR examinations with PC imaging using phantom correction during the study period. From this patient population, 144 patients had PC images of the ascending aorta (AAO), 118 patients had PC images of the main pulmonary artery (MPA), 70 patients had PC images of the right pulmonary artery (RPA), and 61 patients had PC images of the left pulmonary artery (LPA). The patient characteristics and diagnoses are provided in Table 2.

**Table 2:** Patient Characteristics

	Analyzed Patients
**Number of patients**	149

**Median age, years (range)**	17.5 (0.3-69.3)

**Median BSA, m**^2^**(range)**	1.6 (0.3-2.2)

**Tetralogy of Fallot**	36 (25%)

**Cardiomyopathy**	16 (11%)

**Shunt lesions**	15 (10%)

**Conotruncal abnormalities**	13 (9%)

**Single ventricles**	11 (7%)

**Coarctation of the aorta**	10 (7%)

**Valvular regurgitation/stenosis**	28 (19%)

**Great vessel abnormalities**	3 (2%)

**Miscellaneous**	17 (11%)

BSA-Body surface area

### Effect of Phantom Correction

The median effects of phantom correction, presented as absolute change from baseline, are shown in Table 3. Phantom correction resulted in clinically significant changes in 13% to 48% of overall PC measurements in patients with known or suspected heart disease (Table 4). This range of changes is across all measured and calculated quantities. For example, the aortic regurgitation fractions showed the smallest changes, with 13% of measurements changing by > 10% with phantom correction, and the left pulmonary artery flow showed the greatest changes, with 48% of measurements changing by > 0.25 L/min/m^2 ^with phantom correction. Overall, 640 measurements or calculated values were analyzed, and clinically significant changes were found in 197 (31%). As discussed above, cutoff values were assigned prior to analysis to assess how often phantom correction resulted in changes that would have significance on clinical decision-making. There were marked changes in up to 25% of PC measurements (Table 4). Clinically significant or marked changes were generally more common in measurements of output (L/min/m^2^) as compared to regurgitant fractions or flow ratios.

**Table 3:** Overall Effect of Phantom Correction

Variable	N	Median Absolute Change	Range
**MPA flow (L/min/m2)**	118	0.4	0.0-5.0

**AAO flow (L/min/m2)**	144	0.4	0.0-3.5

**RPA flow (L/min/m2)**	70	0.1	0.0-2.6

**LPA flow (L/min/m2)**	61	0.2	0.0-2.1

**Q_P_/Q**_S_	113	0.2	0.0-4.6

**PR fraction (%)**	53	6	0-27

**AR fraction (%)**	24	3	1-14

**Q_P_R fraction (%)**	57	5	0-54

AAO-Ascending Aorta, AR-Aortic Regurgitation, LPA-Left Pulmonary Artery, MPA-Main Pulmonary Artery, PR-Pulmonary Regurgitation, Q_P_-Pulmonary blood flow, Q_S_-Systemic blood flow, Q_P_R-Right pulmonary blood flow as a percent of total pulmonary blood flow, RPA-Right Pulmonary Artery

**Table 4:** Magnitude of Change with Phantom Correction

Variable	N	Clinically Significant Change	Marked Change
**MPA flow (L/min/m2)**	118	50 (42%)	21 (18%)

**AAO flow (L/min/m2)**	144	51 (35%)	15 (10%)

**RPA flow (L/min/m2)**	70	17 (24%)	6 (9%)

**LPA flow (L/min/m2)**	61	29 (48%)	15 (25%)

**Q**_P_**/Q**_S_	113	20 (18%)	7 (6%)

**PR fraction (%)**	53	14 (26%)	1 (2%)

**AR fraction (%)**	24	3 (13%)	0 (0%)

**Q**_P_**R fraction (%)**	57	13 (23%)	6 (11%)

AAO-Ascending Aorta, AR-Aortic Regurgitation, LPA-Left Pulmonary Artery, MPA-Main Pulmonary Artery, PR-Pulmonary Regurgitation, Q_P_-Pulmonary blood flow, Q_S_-Systemic blood flow, Q_P_R-Right pulmonary blood flow as a percent of total pulmonary blood flow, RPA-Right Pulmonary Artery

### Direction of Change with Phantom Correction

Flow measurements in our study increased with phantom correction in some instances and decreased in others (Table 5). AAO flow increased in a significant majority of patients, while LPA flow decreased. Calculated Q_P_/Q_S _ratio and AR fraction decreased more often than increased. For MPA flow, PR fraction, RPA flow, and Q_P_R, there was no significant difference in the direction of change.

**Table 5:** Direction of Change with Phantom Correction

*Measured* *Variables*	Increase with Phantom Correction (N)	Decrease with Phantom Correction (N)	P value
**MPA**	52	66	0.20

**AAO**	109	35	< 0.001

**RPA**	32	38	0.47

**LPA**	20	41	< 0.001

***Calculated*** ***Variables***			

**Q**_P_**/Q**_S_	35	78	< 0.001

**PR**	28	25	0.68

**AR**	6	18	0.01

**Q**_P_**R**	33	24	0.23

AAO-Ascending Aorta, AR-Aortic Regurgitation, LPA-Left Pulmonary Artery, MPA-Main Pulmonary Artery, PR-Pulmonary Regurgitation, Q_P_-Pulmonary blood flow, Q_S_-Systemic blood flow, Q_P_R-Right pulmonary blood flow as a percent of total pulmonary blood flow, RPA-Right Pulmonary Artery

### Effect on a Single Diagnostic Group: Tetralogy of Fallot

There was no significant difference in the effect of phantom correction for any of the measured flows or calculated values for the sub-group of patients with tetralogy of Fallot as compared to the group of analyzed patients as a whole (Table 6). Clinically significant changes in patients with tetralogy of Fallot were present in up to 53% of PC measurements and marked changes in up to 21%.

**Table 6:** Tetralogy of Fallot Patients: Effect of Phantom Correction

Variable	N	Clinically Significant Change	P value v. All Patients	Marked Change	P value v. All Patients
**MPA flow (L/min/m2)**	32	17 (53%)	0.15	6 (19%)	1.00

**AAO flow (L/min/m2)**	34	15 (44%)	0.28	2 (6%)	0.55

**RPA flow (L/min/m2)**	27	7 (26%)	0.64	3 (11%)	0.46

**LPA flow (L/min/m2)**	24	11 (46%)	0.68	5 (21%)	0.64

**Q**_P_**/Q**_S_	31	7 (23%)	0.65	2 (6%)	1.00

**PR fraction (%)**	32	8 (25%)	1.00	1 (3%)	1.00

**AR fraction (%)**	3	0 (0%)	N/S	0 (0%)	N/S

**Q**_P_**R fraction (%)**	22	6 (27%)	0.61	2 (9%)	1.00

AAO-Ascending Aorta, AR-Aortic Regurgitation, LPA-Left Pulmonary Artery, MPA-Main Pulmonary Artery, PR-Pulmonary Regurgitation, Q_P_-Pulmonary blood flow, Q_S_-Systemic blood flow, Q_P_R-Right pulmonary blood flow as a percent of total pulmonary blood flow, RPA-Right Pulmonary Artery

### Q_P_/Q_S _in Patients with Structurally Normal Hearts

**Figure 4:**
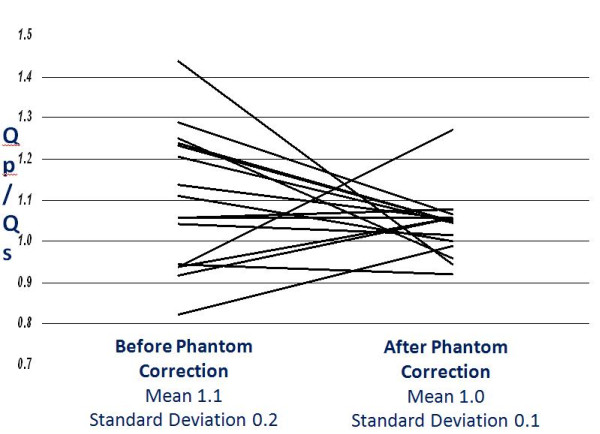
**Q_P_/Q_S _in Patients with Structurally Normal Hearts**. Q_P_-Pulmonary blood flow, Q_S_-Systemic blood flow

Within our patient population, 16 of the 149 patients had structurally normal hearts, based on no shunt lesion and no valvular regurgitation by both echocardiography and CMR. In these patients, Q_P_/Q_S _after phantom correction (1.0 ± 0.1) was closer to 1.0 than before phantom correction (1.1 ± 0.2) (Figure 4, p = 0.016).

### Relationship between Vessel Size and Phantom Correction

**Figure 5:**
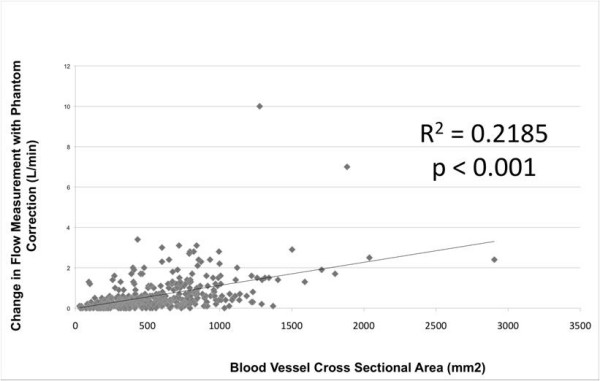
**Flow Correction vs. Blood Vessel Size**.

To assess the effect of blood vessel cross sectional area on the changes in flow measurements with phantom correction, we created a scatter plot of all 392 magnitude flow measurements (AAO, MPA, RPA, and LPA) performed in all patients. There was a small but statistically significant relation between vessel cross sectional area and change in PC flow measurement with phantom correction. Linear regression analysis found that 22% of the changes in PC flow measurements could be attributed to the cross sectional area of the vessel being measured (Figure 5, p < 0.001).

### Relationship between Heart Rate and Phantom Correction

Due to the possibility that a longer R-R interval may allow more time for the velocity offset to accumulate into the flow error, we assessed the effect of heart rate on the changes in flow measurements with phantom correction. Scatterplots were created comparing change in flow measurements in the AAO and MPA to the patient's heart rate during the scan. Using linear regression analysis, we found that heart rate did not contribute significantly to the changes in PC flow measurements in the AAO (R^2 ^= 0.018, p = NS) or MPA (R^2^< 0.001, p = NS).

## Discussion

In our study at a single institution with imaging performed using a single scanning platform, phantom correction often resulted in clinically significant changes in CMR blood flow measurements in patients with known or suspected congenital heart disease. Of 640 total measurements or calculated values that were analyzed, clinically significant changes were found in 31%.

Based on our definitions, which were chosen prior to analysis, 13% to 48% of flow measurements in our study were sufficiently altered by phantom correction enough to potentially alter clinical management. There was no consistent directionality to the changes as a whole, except that AAO flow measurements were generally greater following phantom correction. Miller et al. recently analyzed a smaller group of 31 patients with Tetralogy of Fallot, bicuspid aortic valve, or atrial level shunts, and also found potentially clinically significant and unpredictable variations in PC flow measurements with phantom correction [7].

In the subgroup of 16 patients with structurally normal hearts with an expected Q_P_/Q_S _of 1.0, phantom correction did result in a Q_P_/Q_S _closer to 1.0, similar to what has been reported previously by Chernobelsky et al [8]. In their study of 10 adult volunteers with structurally normal hearts, phantom correction resulted in a change of the measured pulmonary-to-systemic flow ratio (Q_P_/Q_S_) from 1.25 ± 0.20 before correction to 1.05 ± 0.07 after correction.

In addition, the magnitude of phantom correction was strongly correlated with blood vessel cross-sectional area in the study by Chernobelsky [8]. A similar analysis in the current study also demonstrated a relationship between blood vessel cross sectional area and the change in flow with phantom correction, but this relationship only explained 22% of the changes with phantom correction. It is possible that our patients had less laminar flow in the measured vessels, for example in a reconstructed right ventricular outflow tract. The vessels were smaller in the current study compared to the study by Chernobelsky et al.; in addition, flow measurements in the RPA and LPA were analyzed in the current study along with flow in the AAO and MPA.

Our patient population was heterogeneous, varying in diagnoses, age, and size, representing a clinically realistic use of CMR PC measurements in a variety of different patient types. Patients with repaired tetralogy of Fallot made up the largest single group, and subgroup analysis of these patients showed they did not differ significantly from the group as a whole. Our retrospective study design may have introduced a bias due to the selection of patients for PC flow measurements based on clinical criteria.

Technical CMR factors could have impacted the reliability of phantom correction. First, the behavior of the pulse sequence may have been affected by variations in the imaging parameters. Second, there was a time delay of 15 to 20 minutes between PC flow imaging and phantom imaging, which may have allowed for baseline drift. Third, the fluid in the phantom was assumed to be stationary during the image acquisition. We believe that adequate time was allowed in our examination for the fluid to settle, but this was not formally assessed. The phantom images were reviewed for our one patient with a structurally normal heart whose Qp/Qs became less accurate with phantom correction (Figure 4) to look for evidence that the fluid did not settle, but none was found. It is also possible that the distance from isocenter may impact the PC flow error. Chernobelsky et al. [8] looked at anterior-posterior distance from isocenter and did not find a significant effect, but care must be taken to locate the vessel of interest in the z = 0 location in the head-foot direction as well, to minimize this possible source of error.

The most significant limitation to our study is that true flow measurements for these patients are unknown. We found that phantom correction altered flow measurements, but we could not be certain that the corrected measurements are closer to the patient's actual blood flow. These patients did not have flow measurements performed by any other method or a recognized gold standard, to which the CMR PC values with and without phantom correction could be compared. Our subgroup analysis of patients with structurally normal hearts indicated that phantom correction was indeed providing more accurate measurements. However, more work is needed to verify that phantom correction results in more accurate blood flow measurements in patients with abnormal hemodynamic states due to congenital heart disease. An additional study limitation is that we investigated the impact of phantom correction on one MRI platform, and that it is unknown whether these results will be generalizable to other scanners. A multicenter investigation of background phase offset errors using gelatin phantoms has highlighted the potential effect of phase offset errors in CMR on multiple scanning platforms [6].

Based on our findings, it remains our standard protocol to perform phantom correction for each PC flow measurement. While there is considerable time and energy involved in acquiring additional images and in analysis, we feel that, based on the best available information, the correction of PC flow measurements with phantom imaging yields more accurate results in our laboratory.

## Conclusions

Phantom correction often results in clinically significant changes in CMR blood flow measurements in patients with known or suspected congenital heart disease. Phantom correction can result in increased or decreased flow measurements, with no consistent directionality to the changes as a whole. In laboratories performing clinical CMR with suspected phase offset errors of significance, the routine use of phantom correction for PC flow measurements should be considered.

## List of Abbreviations Used

AAO: ascending aorta; AR: aortic regurgitation; CHD: congenital heart disease; CMR: cardiac magnetic resonance; LPA: left pulmonary artery; MPA: main pulmonary artery; PC: phase-contrast; PR: pulmonary regurgitation; Q_P_/Q_S_: pulmonary-to-systemic flow ratio; Q_P_R: percent flow to the right pulmonary artery; ROI: region of interest; RPA: right pulmonary artery.

## Competing interests

The authors declare that they have no competing interests.

## Authors' contributions

BJH performed the statistical analysis and drafted the manuscript

BFP participated in the design of the study and helped to draft the manuscript

WWL conceived of the study, participated in the design of the study, and helped to draft the manuscript.
